# The Success of Ovulation Induction with Letrozole and Gonadotropins in Obese and Nonobese Women: A Study from a Tertiary Center

**DOI:** 10.1155/2022/1931716

**Published:** 2022-06-17

**Authors:** Vaidyanathan Gowri, Arwa Al-Amri, Thikra Mohammed Abdulrahman Almamari, Maha Al Khaduri, Sanjay Jaju

**Affiliations:** ^1^Department of Obst. Gynecology, Sultan Qaboos University and Hospital, Muscat, Oman; ^2^Sultan Qaboos University, Oman; ^3^Department of Family Medicine and Community Health, Sultan Qaboos University, Muscat, Oman, Sultan Qaboos University, Muscat, Oman

## Abstract

**Background:**

Letrozole, an aromatase inhibitor, is suggested as a first-line drug for ovulation induction in women with polycystic ovary syndrome (PCOS) especially in obese women. Letrozole has also been used in women with unexplained infertility with similar rates of success to clomiphene. However, literature on letrozole and gonadotropins in obese and nonobese women is sparse. Hence, this study was done to assess the success of ovulation induction (OI) with letrozole plus follicle stimulating hormone (FSH) in obese (BMI ≥ 30 kg/m^2^) and nonobese women (BMI < 30 kg/m^2^).

**Methods:**

A retrospective descriptive cohort study was conducted involving 135 women who underwent OI with letrozole plus follicle stimulating hormone therapy and either timed intercourse or intrauterine insemination. The data was collected from the hospital information system, including the age, body mass index, the type of infertility, number of induction cycles with letrozole, number of gonadotropin injections, and the pregnancy occurrence following treatment. SPSS was used to analyze the data.

**Results:**

There were 135 women who used FSH injections along with letrozole. Of this, 28.5% obese women got pregnant compared to 29.2% nonobese women, but this did not attain statistical significance (*P* = 0.75). About 70% of obese women and 57% on nonobese women had polycystic ovarian syndrome. The median number of FSH injections was six, and the interquartile range was 3 to 11.

**Conclusion:**

Of the 135 women undergoing letrozole and FSH, there was almost an equal probability of pregnancy in the obese group (BMI ≥ 30 kg/m^2^) and nonobese women.

## 1. Background

Obesity is considered as one of the most frequently observed risk factors for infertility in both males and females as well, as it interferes with the success of the treatment of fertility [1]. The prevalence of obesity parallels increased industrial development which, in the Arabian Gulf, is related to a significant growth in income from rich deposits of oil reserves and resulting rapid urbanization and improved living conditions [2].

Higher body mass index is associated with infertility, especially ovulatory disorders [3]. Obese women under treatment for infertility may face additional problems, such as the need for higher doses of drugs to induce/stimulate ovulation, oocyte morphological changes, reduction in fertilization and implantation rates, and embryo quality [4].

Letrozole and clomiphene citrate are commonly used drugs for ovulation induction with or without gonadotropins in anovulatory women especially polycystic ovarian syndrome. Letrozole, a short half-life aromatase inhibitor (45 hours), has shown more successful ovulation induction in polycystic ovarian syndrome (PCOS) patients. Initially clomiphene (CC) and letrozole were found to give similar ovulatory and pregnancy rates [5], but a randomized trial [6] and a meta-analysis of 14 trials in nearly 3000 anovulatory women with PCOS [7] suggest that letrozole therapy results in higher live birth rates compared with clomiphene therapy. Letrozole is considered as an attractive option for oral ovulation induction because the effect of letrozole on estrogen receptors is indirect which is thought to contribute to less antiestrogenic side effects than CC which directly acts on estrogen receptors [8]. In cases of poor follicular response, low doses of FSH are given as a cotherapy with letrozole to enhance follicular development and maturity. According to McKnight et al., letrozole has been found to be an effective oral ovulation induction therapy and a viable therapy for obese women when combined with gonadotropins [9]. Letrozole cotreatment with gonadotropins was found to cause a higher incidence of monofollicular growth which is an advantage that reduces the risks of hyperstimulation effect of ovulation induction therapy [10]. Letrozole was also used in unexplained infertility and found to be as effective as clomiphene citrate with reduced multiple births [11]. However, a combination of letrozole and gonadotropins has not been studied extensively either in PCOS patients or unexplained infertility, in relation to obesity. Therefore, the goal of this study was to assess the success of ovulation induction with letrozole combined with FSH in obese and nonobese women. The main intention was to study the impact of obesity on fertility outcome, when FSH was used along with letrozole.

## 2. Methods

This was a retrospective study conducted at Sultan Qaboos University Hospital (SQUH), a tertiary care center in Oman. It was ethically approved by the Medical and Research Ethics Committee in the College of Medicine and Health Sciences. The list of women, who underwent ovulation induction (OI) with letrozole-containing therapy (letrozole with gonadotropin injection, follicle stimulating hormone) between January 2015 and February 2017, was acquired from the infertility clinic registry. Letrozole was prescribed at a dose of 5 mg from cycle day 3 to cycle day 7 and follicle stimulating hormone on days 5, 7, and 9 of the cycle (recombinant injection r-hFSH, Gonal-F®; Serono), and follicular monitoring started from day 9. Women with mild male factor infertility were included. When there was a dominant follicle, women either had timed intercourse after human chorionic gonadotropin trigger or underwent intrauterine insemination (IUI). Medical records were examined to obtain the demographic data that included information about age, weight, and height used at the time closest to the time starting OI therapy. Patients were then classified into two main groups depending on body mass index (BMI): nonobese group with BMI < 30 kg/m^2^ and obese group with BMI ≥ 30 kg/m^2^. For BMI calculation, measured height and weight were used. Other data included the type of infertility, number of induction cycles with letrozole, number of gonadotropin injections, and pregnancy achievement following this treatment.

Exclusion criteria included women who got pregnant spontaneously or with in vitro fertilization (IVF) and women with insufficient data about body mass index (BMI).

To analyze the data, Statistical Package of Social Sciences (SPSS) program version 23.0 was used to create a database of all variables that were set. One-sample Kolmogorov-Smirnov test (K-S test) was used to verify if a continuous variable obeys the normal distribution pattern. As the study variable did not follow the normal distribution pattern and showed many outliers, which are variables too distant from other values, it was found that the median would be appropriate to be used here than mean because it would get highly affected by the outliers.

## 3. Results

Results showed that one hundred and thirty-five patients with a variety of infertility diagnoses underwent 380 letrozole-containing therapy cycles and met the study criteria. Of the 135 women enrolled in the study, 65 (48%) were nonobese with BMI less than 30 kg/m^2^ and the remaining 70 (52%) women were obese with BMI more than or equal to 30 kg/m^2^. In the nonobese group, the median age was 30 years and the interquartile range (IQR) was 26 years to 35 years. For the obese group, the median age was 31 and the IQR range was 27 years to 34 years, (Table 1). Of the 135 women, 61% had PCOS and the remaining was unexplained infertility.

In the nonobese group, 44.6% had primary infertility, and in the obese group, 37% had primary infertility. Both BMI groups showed a higher prevalence of secondary infertility than primary infertility but the difference was not significant (*P* = 0.36).

After combining FSH injection with letrozole for enhancing follicular development and maturation in these patients, a total of 28.5% of women with BMI ≥ 30 kg/m^2^ and 29.2% of women with BMI < 30 kg/m^2^ achieved pregnancy with no significant difference (*P* = 0.75) (Table 2). Of the total 39 women who got pregnant, 31 were in the PCOS group (Table 3). In the unexplained group, about 15% got pregnant with equal distribution in the obese and nonobese group. There were three women with multiple pregnancies in the obese PCOS group and one woman with multiple pregnancies in the unexplained, nonobese group.

The mean number of cycles was three for each patient. The number of FSH injections per patient was a median of 6 with an interquartile range (IQR) 3-11.

There were 12 miscarriages (some women had more than one miscarriage) in total in the nonobese women and 11 miscarriages in the obese women.

## 4. Discussion

In this study, there were 135 women undergoing letrozole-containing treatment for infertility. There were no significant differences in age, number of induction cycles, and FSH injections between the two groups of BMI 30 kg/m^2^ and ≤30 kg/m^2^. Both BMI groups showed a higher prevalence of secondary infertility than primary infertility but the difference was not significant (*P* = 0.36). About 61% women had PCOS, and the remaining was unexplained infertility.

Women with BMI 30 were more likely to conceive with letrozole and FSH according to reports by McKnight et al. [9]. It is hypothesized that letrozole decreases the conversion of testosterone to estrogen peripherally, thus decreasing pituitary inhibition, and promotes normal secretion of gonadotropins. However, in view of obesity, these women needed more FSH. The dose of FSH injections did not differ significantly between obese and nonobese women in our study. According to Kaya et al., increased BMI was associated with the increase in FSH requirement and a longer period of ovarian stimulation that could result in follicle development [12]. The number of follicles also did not differ significantly between the obese and nonobese women in our study.

Yun et al. studied minimal stimulation protocol using clomiphene or letrozole and gonadotropins and found significantly less ovarian hyperstimulation syndrome in the letrozole group with comparable pregnancy rates, though obesity was not mentioned in their study [13]. However, they did not include women with PCOS in their study. As compared with clomiphene, letrozole was associated with higher live-birth and ovulation rates among infertile women with the polycystic ovary syndrome according to Legro et al., but the mean BMI in their study was 35 [6].

We studied both the PCOS and unexplained groups with the main focus on obesity, with a BMI > 30. In view of the small sample size, it was not possible to stratify into various classes of obesity and analyze the data. The number of non-PCOS women was also very less in this study. Stratifying into various obesity groups and including only non-PCOS women are something worth looking into in the future.

In conclusion, in the small number of women we studied, letrozole and FSH were equally effective and there was not a single woman with ovarian hyperstimulation.

### 4.1. What Is Known about This Topic

Letrozole is a first-line drug in women with PCOS.

Letrozole and gonadotropin combination is used for ovulation induction for IUI.

### 4.2. What This Study Adds

Letrozole with FSH had similar pregnancy rates in obese and nonobese PCOS women.

The pregnancy rate was similar in obese and nonobese women with unexplained infertility but the numbers studied were small.

## Figures and Tables

**Table 1:** Demographic and clinical data for women undergoing ovulation induction with letrozole+FSH-containing therapy.

Variable	BMI < 30 kg/m^2^	BMI ≥ 30 kg/m^2^	*P* value
Age	30 (26-35)	31 (27-34)	0.79
Number of women	65	70	
Number of induction cycles	3 (1-3)	2 (1-4)	
Number of pregnancies	19	20	0.95

**Table 2:** Number of follicles and FSH injections used in each group.

		BMI groups		Chi-square *P* value
		<30	≥30	
FSH inj per cycle	1-6 inj	51	58	NS
	7-12 inj	8	8	
	≥13 inj	6	4	
Number of follicles per cycle	≤3	33	34	NS
	4-6	20	21	
	>6	12	15	
Pregnant	Yes	19	20	NS
	No	46	50	

**Table 3:** Pregnancy in PCOS and unexplained infertility.

	Nonobese < 30	Obese ≥ 30
PCOS	37	46
Unexplained	28	24
Pregnant PCOS	15	16
Pregnant unexplained infertility	4	4

## Data Availability

Data is available on request.
